# Implementation of Medical Therapy in Different Stages of Heart Failure with Reduced Ejection Fraction: An Analysis of the VIENNA-HF Registry

**DOI:** 10.3390/biomedicines13081846

**Published:** 2025-07-30

**Authors:** Noel G. Panagiotides, Annika Weidenhammer, Suriya Prausmüller, Marc Stadler, Georg Spinka, Gregor Heitzinger, Henrike Arfsten, Guido Strunk, Philipp E. Bartko, Georg Goliasch, Christian Hengstenberg, Martin Hülsmann, Noemi Pavo

**Affiliations:** 1 Department of Internal Medicine II, Clinical Division of Cardiology, Medical University of Vienna, 1090 Vienna, Austria; noel.panagiotides@meduniwien.ac.at (N.G.P.);; 2 Complexity Research, 1050 Vienna, Austria

**Keywords:** advanced heart failure, HFrEF, phenotypes, guideline directed medical therapy, up-titration limiting factors, adverse events

## Abstract

**Background/Objectives:** Real-world evidence shows alarmingly suboptimal utilization of guideline directed medical therapy (GDMT) in heart failure with reduced ejection fraction (HFrEF). One of the barriers of GDMT implementation appears to be concerns about the potential development of drug-related adverse events (AEs), particularly in high-risk patients. This study aimed to evaluate whether advanced HFrEF (AHF) patients can be up-titrated safely and whether AHF predisposes individuals to the occurrence of putatively drug-related AEs. **Methods:** A total of 373 HFrEF patients with documented baseline, 2 months, and 12 months visits were analyzed for utilization and target dosages (TDs) of HF drugs. Successful up-titration and AEs were evaluated for different stages of HF reflected by N-terminal pro-B type natriuretic peptide (NT-proBNP) (<1000 pg/mL, 1000–2000 pg/mL, >2000 pg/mL). **Results:** A stepwise increase in HF medications was observed for all drug classes during follow-up. At 12 months, 73%, 75%, 62%, 86%, and 45% of patients received ≥90% of TDs of beta-blockers (BBs), renin–angiotensin system inhibitors (RASis), mineralocorticoid receptor antagonists (MRAs), sodium–glucose cotransporter-2 inhibitors (SGLT2 i), and triple-therapy, respectively. Predictors of successful up-titration in logistic regression were baseline HF drug TDs, estimated glomerular filtration rate (eGFR), and potassium, but not NT-proBNP or age. The development of AEs was rare, with hyperkalemia as the most common event (34% at 12 months). AEs were comparable in all stages of HF. However, the development of hyperkalemia was more frequent in patients with higher NT-proBNP and also accounted for most cases of incomplete up-titration. **Conclusions:** This study suggests that with dedicated protocols and frequent visits, GDMT can be successfully implemented across all stages of HFrEF, including patients with AHF.

## 1. Introduction

Heart failure (HF) remains a leading cause of morbidity and mortality worldwide [1,2]. Despite transformative advances in HF therapies, their implementation and up-titration in clinical practice lags alarmingly behind. Only 22% of HF patients receive some form of triple-therapy, and a mere 1% reach the recommended target doses (TDs) of all essential medications [3].

Previously reported barriers of guideline directed medical therapy (GDMT) implementation include therapeutic inertia, misperceptions about a “clinically stable” status, potential biases against older, female or co-morbid patients, and concerns over therapy-related adverse events (AEs) such as hypotension, impaired renal function, or hyperkalemia [3,4,5,6]. “Clinically stable” is often equated with acceptable symptoms or vital signs, which may lead to missed opportunities for therapy intensification despite ongoing subclinical disease progression. This misconception can create a false sense of reassurance and delay essential treatment optimization. Furthermore, women with heart failure with reduced ejection fraction (HFrEF) are consistently undertreated compared with men and receive lower rates and intensities of GDMT over time [7]. Physicians may also underestimate symptom burden in women, contributing to less aggressive treatment and reinforcing existing disparities in care [8]. Clinicians tend to most frequently underuse GDMT in advanced HF (AHF) patients due to a lack of strong evidence in this patient population and concerns about drug-related AEs, while these patients potentially benefit most [9].

This study aimed to (i) explore whether AHF patients can be up-titrated safely with HF medications and (ii) assess the development of drug (and disease)-related AEs during medical up-titration in a dedicated HF outpatient unit in a tertiary care center.

## 2. Materials and Methods

### 2.1. Patient Population

Since 2015, a HFrEF registry at the Vienna General Hospital—an affiliated academic institution—includes chronic HFrEF outpatients defined by a history of documented left ventricular ejection fraction (LVEF) of ≤35% by echocardiography and a history of significantly elevated N-terminal pro-B type natriuretic peptide (NT-proBNP) levels > 1500 pg/mL. The inclusion criteria for this study therefore required both a confirmed LVEF ≤ 35% by echocardiography and NT-proBNP >1500 pg/mL prior to inclusion into the register. Clinical data, patient history, cardiac imaging results, medications, and laboratory values are routinely documented at every visit. Comorbidities such as chronic kidney disease, diabetes, and atrial fibrillation were systematically recorded and classified based on the International Classification of Diseases criteria. For this study, the following patients were selected: patients between January 2015 and December 2023 were analyzed, who completed a baseline (BL), short-term (2 ± 1 months, 2 M visit), and long-term follow-up visit (12 ± 6 months, 12 M visit). Patients with insufficient follow-up were excluded. Echocardiographic assessments were performed according to standard protocols in our institution and were repeated as clinically indicated during follow-up. The investigations were conducted in strict adherence to the principles outlined in the Declaration of Helsinki and received institutional ethics committee approval (EK1612/2015). All participants provided written informed consent.

### 2.2. Patient and Public Involvement

Patients were not involved in the design, conduct, reporting, or dissemination plans of this research. This study was based on a prospective heart failure registry capturing routine clinical care at a tertiary outpatient heart failure clinic. While patients did not directly contribute to the study design, the research question was shaped by a clear clinical need to address gaps in evidence around the tolerability and implementation of heart failure therapies, particularly in advanced disease stages. The findings are intended to inform future clinical practice and improve therapeutic outcomes for patients with heart failure.

### 2.3. Assessing GDMT in HF

Precise use and exact dosage of HF medications, i.e., beta-blockers (BBs), renin–angiotensin system inhibitors (RASis), mineralocorticoid receptor antagonists (MRAs) and sodium–glucose cotransporter-2 inhibitors (SGLT2i), were documented at each visit. In this study, implementation of therapy refers to active initiation and up-titration of each medication class by the treating physician, with documentation of dose changes, patient adherence, and any reasons for discontinuation or non-initiation (e.g., adverse events or intolerance). For comparability, HF medication dosages were expressed as a percentage of the recommended TDs. Non-receipt of a specific drug was recorded as 0%. Additionally, triple-therapy intensity was assessed by calculating the mean percentage of the TD for BB, RASi, and MRA [$triple-therapy\;TD\%=\frac{1}{3}\times (BB\;TD\%+RASi\;TD\%+MRA\;TD\%)$] [10,11]. Dosing optimization was performed according to the ESC guidelines, with medication adjustments made at each visit or as clinically indicated. SGLT2i data in this paper was only analyzed for patients enrolled after 2021, in alignment with the ESC recommendations for SGLT2i use.

### 2.4. Definition of Clinical Factors Limiting GDMT Up-Titration—Adverse Events

The presence of clinical factors potentially limiting GDMT up-titration/AEs related to HF treatment were assessed at each visit. AEs were defined according to the 2021 ESC HF-guidelines [10]. The most common clinical AEs were defined as follows: bradycardia, indicated by a resting heart rate (RHR) of <50 beats per minute (bpm); asymptomatic hypotension, identified by an office systolic blood pressure (SBP) of <90 mmHg without symptoms; symptomatic hypotension, identified by an office SBP of <90 mmHg with symptoms; impaired renal function, indicated by an eGFR of <30 mL/min/1.73 m^2^; and hyperkalemia (HK), indicated by a serum potassium (K) level of >5.0 mmol/L or >5.5 mmol/L. AEs that were not present as the initial visit but developed at follow-up (FUP) during up-titration were termed new AEs. In case HF drugs were not up-titrated beyond 50% TD, medical records were investigated to identify the reason for no up-titration.

### 2.5. Statistical Analysis

To describe the patient population, baseline characteristics are presented. Continuous data are expressed as median and interquartile range (IQR), categorial data as counts and percentages. To compare the BL characteristics according to up-titrational success, counts were analyzed by the 2-sided Fisher’s exact test, and continuous variables by the Kruskal–Wallis test.

To assess the success of GDMT up-titration, TDs were compared by the Friedman test across all timepoints (paired, non-parametric test). To identify specific differences, post hoc analysis was performed using pairwise Wilcoxon Signed-Rank Tests (paired, non-parametric test). To correct for multiple comparisons, Bonferroni correction was applied. For visualization, TDs were summarized in four clinically relevant groups, i.e., zero (0%), low (>0–<50%), medium (≥50–<90%), and high (≥90%) TDs [11].

To identify factors associated with up-titrational success, i.e., TDs of ≥90% at 12-month follow-up, a logistic bootstrap regression model with stepwise forward selection (*p* ≤ 0.05 for inclusion) as an exploratory method was used. Potential influencing factors considered included age, sex, body mass index (BMI), NYHA class, SBP, HR, K, eGFR, blood urea nitrogen (BUN), sodium, butyrylcholinesterase (BChE), GOT, GPT, GGT, bilirubin, transferrin saturation (TSAT), hemoglobin, triglycerides, C-reactive protein (CRP), NT-proBNP, number of comorbidities, and baseline TDs. The selection process followed the method described by Harrell [12]. It accounts for potential non-linear associations and initially excludes variables with weak bivariate correlations (*p* > 0.2). Variables selected in fewer than 40% of 500 bootstrap samples using forward selection were then excluded step by step. A final logistic regression with forward selection was performed on the remaining predictors. The results are reported in units of standard deviation. Alternative selection methods were tested by using continuous TD values, case-by-case exclusion of missing data or imputation by the mean, and consideration of first-order intercorrelations. These alternatives produced qualitatively consistent results for the key variables (*p* ≤ 0.01).

To visualize the main results of the logistic regression, HF TD groups were displayed for clinically relevant variables, i.e., NT-proBNP, eGFR, age, sex, BMI, and comorbidity burden. In this analysis, trends for GDMT up-titration were analyzed between the strata by the Jonckheere–Terpstra test (unpaired, non-parametric test, which calculates *p* for trend). Sex differences were compared via the Kruskal–Wallis H test (unpaired, non-parametric test).

The prevalence of AEs was displayed as percentages at different timepoints. AEs were compared by the McNemar–Bowker test (paired, categorical test) between timepoints. For an explorative analysis, the distribution of new AEs after 12 months according to subgroups (NT-proBNP, eGFR, age, BMI, sex, and number of comorbidities) was compared by the 2-sided Fisher’s exact test (unpaired, categorial test).

IBM SPSS 25.0 and GraphPad Prism 9 were used to perform statistical analyses and create the figures. GChaos 31.3 statistical software written in C++ by one of the authors (GS) was used for the bootstrap selection process. A two-tailed *p*-value < 0.05 was deemed statistically significant. In the case of multiple testing, *p*-values were adjusted by Bonferroni correction.

## 3. Results

### 3.1. Study Population

Baseline characteristics for the total cohort (n = 373) are displayed in Table 1. The median age was 62 years (IQR: 50–72); 23.3% of patients were women. A total of 7.8%, 51.6%, and 40.6% of patients were NYHA class I, II, and III/IV; median NT-proBNP was 2363 pg/mL (IQR: 1014–5009).

### 3.2. Up-Titration of HF Medication

**Figure 1:**
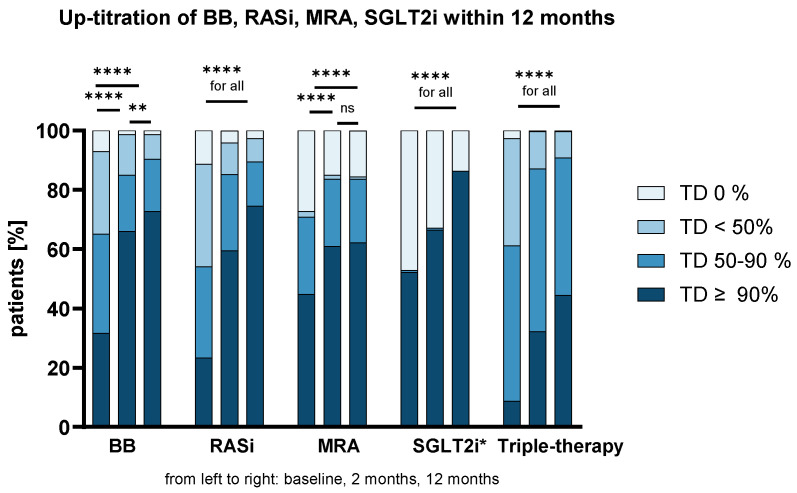
Up-titration of HF medication in a HFrEF cohort at a tertiary care center within 12 months. The percentage of patients with the achieved target dose (TD) of beta-blockers (BBs), renin–angiotensin system inhibitors (RASis), mineralocorticoid receptor antagonists (MRAs), sodium–glucose co-transporter 2 inhibitors (SGLT2i*, since 2021), and triple-therapy are shown at baseline, 2 months, and 12 months. Data was compared by the Friedman test and post hoc analysis using Bonferroni correction. ns = not significant; ** *p* < 0.01; **** *p* < 0.0001.

The achieved TDs for all HF drug classes at BL, 2 M, and 12 M are shown in Figure 1. A significant increase in TDs could be observed for all drug classes during FUP (*p* < 0.001 for comparisons between BL vs. 2 M and BL vs. 12 M for the continuous variable of TDs). The majority of patients received ≥50% and ≥90% of the recommended TDs at 12 M (for ≥50%: 90%, 90%, 84%, 86%, and 91%; and for ≥90%: 73%, 75%, 62%, 86%, and 45% for BB, RASi, MRA, SGLT2i, and triple-therapy, respectively).

### 3.3. Predictors for Successful Up-Titration—Impact of Disease Severity and Patient Characteristics

Multiple linear regression was conducted to determine the predictors of achieved TD of triple-therapy at 12 months (Table 2). The overall model was statistically significant (R^2^ = 0.150, Cox and Snell). Significant predictors were eGFR (ß = 0.679, *p* < 0.001), serum potassium (ß = −0.240, *p* = 0.048), and triple-therapy at baseline (ß = 0.523, *p* < 0.001).

**Figure 2:**
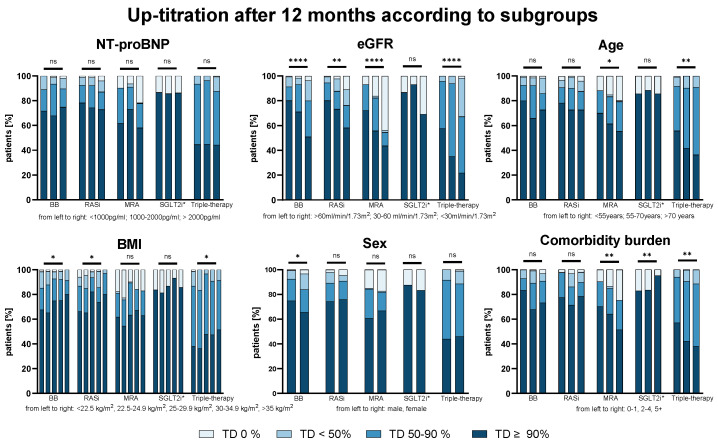
The up-titration of HF medication based on the subgroups after 12 months. The percentage of patients with the achieved target dose (TD) of beta-blockers (BBs), renin–angiotensin system inhibitors (RASis), mineralocorticoid receptor antagonists (MRAs), sodium–glucose co-transporter 2 inhibitors (SGLT2i*, since 2021), and triple-therapy are shown after 12 M according to NT-proBNP, eGFR, age, BMI, sex, and comorbidity burden. Data trends were analyzed by the Jonckheere–Terpstra test; sex was evaluated by the Kruskal–Wallis H test. ns = not significant; * *p* < 0.05; ** *p* < 0.01; **** *p* < 0.0001.

Successful up-titration was independent from heart failure severity reflected by NT-proBNP or NYHA class, but also age, comorbidity burden, sex, and BMI. Figure 2 displays the main findings graphically. However, a consistent trend for lower GDMT was observable with increasingly impaired renal function (BB: *p* < 0.001, RASi: *p* = 0.001, MRA: *p* < 0.001, SGLT2i: *p* = ns, triple-therapy: *p* < 0.001).

### 3.4. Frequency of AEs—Impact of Disease Severity and Patient Characteristics

**Figure 3:**
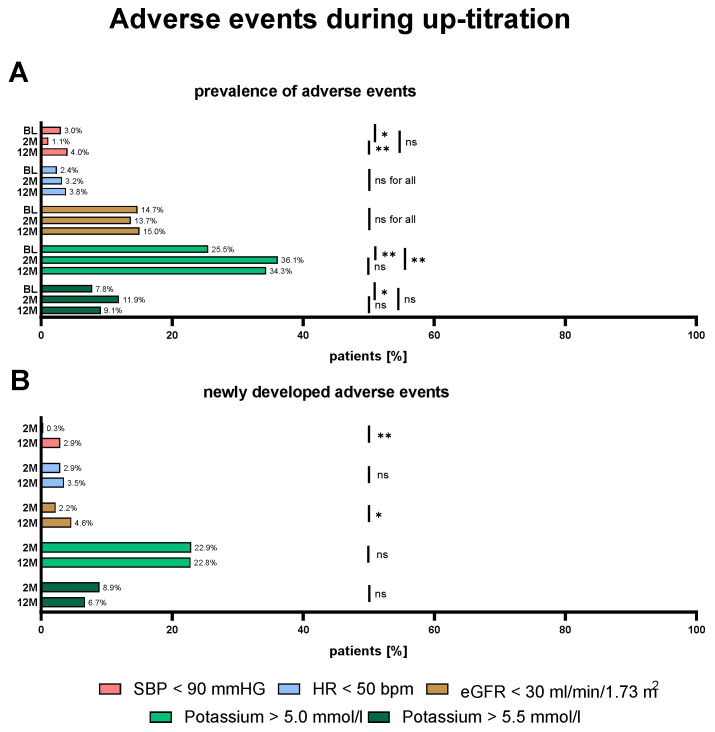
Prevalence of adverse events (AEs) and newly developed AEs. The percentage of patients who developed AEs at baseline (BL), 2 months (2 M), or 12 months (12 M) (**A**) and the percentage of patients with newly developed AEs (**B**) at 2 M or 12 M are shown. Data was compared by the McNemar–Bowker test. ns = not significant; * *p* < 0.05; ** *p* < 0.01.

Figure 3 shows the prevalence and development of AEs at BL, 2 M, and 12 M. At baseline, 3% of patients exhibited hypotension, 2.4% bradycardia, 14.7% impaired renal function, and 25.5% mild (K > 5.0 mmol/L) and 7.8% significant (K > 5.5 mmol/L) hyperkalemia. The development of new AEs was generally infrequent, with less than 5% of cases experiencing hypotension, bradycardia, and renal impairment at 2 M and 12 M. The prevalence of hypotension, bradycardia, and impaired renal function remained similar during medical up-titration. New cases of hyperkalemia were the most frequent new AE and developed in 22.9% and 22.8% (K > 5.0 mmol/L) and 8.9% and 6.7% (K > 5.5 mmol/L) of cases after 2 and 12 months.

**Figure 4:**
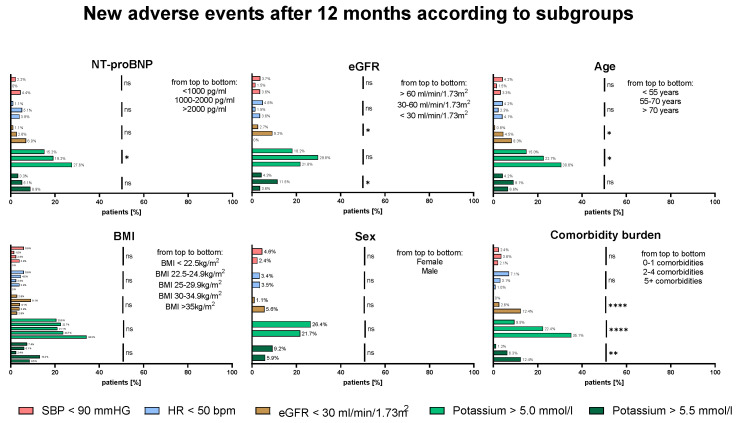
The distribution of newly developed adverse events (AEs) after 12 months according to subgroups. The percentage of patients who developed new AEs at 12 M according to NT-proBNP, eGFR, age, BMI, sex, and comorbidity burden are shown. Data was compared by Fisher’s exact test. ns = not significant; * *p* < 0.05; ** *p* < 0.01; **** *p* < 0.0001.

Figure 4 shows an explorative analysis for the development of HF drug-related AEs at 12 M for clinically relevant patient subgroups. New AEs such as hypotension, bradycardia, or impaired renal function were equally distributed across NT-proBNP strata, sex, or BMI (*p* = ns for all). Only the development of hyperkalemia (K > 5.0 mmol/L at 12 M) increased with higher NT-proBNP (15.2% vs. 19.2% vs. 27.6%, *p* = 0.045). Generally, the development of hyperkalemia (K > 5.0 mmol/L at 12 M) and impaired renal function (eGFR < 30 mL/min/1.73 m^2^) was associated with higher age and increased comorbidity burden. Both higher age and increased comorbidity burden predispose individuals to worse baseline renal function.

### 3.5. Reasons for No Up-Titration (TD ≤ 50%) at 12 M

Table 3 shows the reason for maintaining suboptimal TDs. After 12 months, 23%, 22%, and 38% of patients could not be up-titrated beyond 50% of the recommended TD. Likely causes could be identified for 92%, 91%, and 75% of BB, RASi, and MRA cases. The most common causes were bradycardia and hypotension for BBs (accounting for 66% in total), hypotension and hyperkalemia for RASi (in total accounting for 61%), and hyperkalemia with or without impaired renal function for MRAs (in total accounting for 58%). Among all HFrEF medications, MRAs were most likely to remain at suboptimal dosages and showed the highest number of cases where no reason could be identified. Hyperkalemia accounted for a total of 31% and 43% of cases for suboptimal dosages of RASi and MRA.

## 4. Discussion

This is the first study to investigate differences in GDMT implementation according to the severity of heart failure and to deliver data on the development of AEs during up-titration of GDMT in AHF in a tertiary care outpatient HF center. GDMT implementation was good, whereby 91% of patients achieved ≥50% of the TDs in all recommended drug classes at 1-year. GDMT up-titration could be successfully achieved regardless of NT-proBNP strata or sex. Despite successful up-titration, new AEs remained rare, with only 4%, 4%, 5%, and 7% of patients developing bradycardia, hypotension, severe renal impairment (eGFR < 30 mL/min/1.73 m^2^), and severe hyperkalemia (K > 5.5 mmol/L). A trend for increasing number of AEs and lower average dosages in GDMT was observed in patients with older age, worse baseline renal function, and higher comorbidity burden.

### 4.1. GDMT Evidence in AHF

In a recent universal definition of heart failure from multiple HF societies, AHF is defined by several key characteristics, such as severe symptoms or symptoms at rest, recurrent HF hospitalizations despite GDMT, and requirement of advanced therapies such as mechanical circulatory support [13]. Most importantly, intolerance to up-titration of GDMT is part of this definition.

Other definitions, such as the updated HFA-ESC definition, refer to symptoms, NT-proBNP, and cardiac dysfunction but do not mention the inability of up-titration [14].

There is no established consensus on when to halt GDMT up-titration or how to accurately define true medication intolerance. Moreover, evidence on the efficacy or futility of HF medications in patients with AHF is scarce, leaving clinicians with limited guidance for optimizing treatment in this high-risk population.

Trials in high-risk AHF patients, such as CONSENSUS, COPERNICUS, and RALES, demonstrated the efficacy of ACEi, BBs, and MRAs in this population, with significant reductions in mortality and the combined risk of death or HF hospitalization [15,16,17]. Similarly, in PARADIGM-HF, ARNI showed consistent efficacy regardless of NT-proBNP levels, including those above and below the median of 1631 pg/mL, although patients, in general, were lower risk [18].

### 4.2. GDMT Implementation and Clinical Patient Profiles in AHF

Global registries reveal suboptimal use of GDMT in HFrEF, while the STRONG-HF study demonstrated better results after acute heart failure [3,6,11,18,19,20]. For example, in the US CHAMP-HF Registry, only 28%, 17%, 14%, and 77% of patients achieved TDs for BB, ACEi/ARB, ARNI, and MRA, with just 1.1% on triple-therapy at TD [3]. Importantly, the implementation of GDMT was lower in non-specialist or community-based practices compared to cardiology or HF specialty clinics [3]. European data from CHECK-HF show similar results [19].

In our study, the up-titration of all four pillars of GDMT was successfully achieved across all heart failure risk groups, whereby 73%, 75%, 62%, 86%, and 45% of patients received ≥90% of TDs of BB, RASi, MRA, SGLT2i, and triple-therapy, respectively. TDs were consistent in the low-risk group with median NT-proBNP 508 pg/mL [378–707], the intermediate-risk group with median NT-proBNP 1430 pg/mL [1216–1719], corresponding to a PARADIGM-HF cohort, and the high-risk group with median NT-proBNP 4740 pg/mL [3199–8397], corresponding to a typical cohort of patients treated with a ventricular assist device [21].

### 4.3. Characteristics Predisposing Patients to Suboptimal GDMT

Patients with more severe heart failure, greater symptoms, or comorbidities often receive lower doses of GDMT. Studies consistently show that factors like older age, higher NYHA-class, greater NT-proBNP levels, low blood pressure, female sex, poor kidney function, and the presence of other comorbidities cause physicians to refrain from the up-titration of GDMT [3,4,6,19,22,23]. Such caution may be potentially harmful, as accumulating evidence demonstrates that therapies including SGLT2i, ARNI, and vericiguat provide significant benefits even in older patients, those with CKD, and individuals with advanced heart failure symptoms (NYHA class III/IV) [24].

In the present dataset, after structured up-titration, no bias regarding GDMT implementation based on sex could be observed. Most importantly, GDMT implementation was comparably successful between NT-proBNP strata, indicating that up-titration is feasible and worth pursuing for severe disease.

In line with previous reports besides eGFR, older age, lower BMI, and high comorbidity burden were indeed associated with somewhat worse implementation of GDMT, based on univariate analysis. In multivariate analysis, kidney function and potassium remained significant, indicating that the influence of age, BMI, and comorbidities is largely mediated through eGFR.

Notably, in a logistic regression model, NT-proBNP did not predict up-titration at follow-up.

### 4.4. Development of Adverse Events During Up-Titration

The presumably largest limiting factor for GDMT implementation is the expectation of development of HF drug-related AEs. However, data on AEs during up-titration in AHF remains scarce, and factors limiting GDMT are often underreported or poorly documented in studies and registries. Notably, the achievement of high TDs in registries is far less common than observed in this study. In BIOSTAT-CHF, drug intolerance was cited as a reason for not achieving target doses in 22% of BB patients and 26% of ACEi/ARB, though in most cases, the reasons were unknown or unrecorded [6]. A secondary analysis of GUIDE-IT found that common reasons for avoiding up-titration included the perception of “clinically stable” status or being “already at maximally tolerated therapy” [4]. GUIDE-IT reported an overall low rate of symptomatic hypotension (2%), symptomatic bradycardia (0%), hyperkalemia (2.5%), and worsening renal function (3.6%), but did not differentiate between disease severity [25].

A recent meta-analysis highlighted the misperception of the development of AEs related to HF therapy. The study included landmark cardiovascular outcome trials with forced up-titration in HFrEF and investigated the occurrence of AEs between different HF drugs and placebo [26]. Almost all clinically relevant AEs were rare and occurred at similar rates in both treatment and control groups. This indicates that events such as drops in blood pressure, worsening renal function, or hyperkalemia are not primarily attributable to specific therapies but probably rather reflect the underlying risks associated with HF itself. The overall frequency of drug-related AEs in these trials was low [26]. It can be presumed that these underlying risks are a function of disease stages.

This study reinforces these findings showing that the development of HF drug-attributable AEs during medical up-titration is low and that up-titration can be achieved in a dedicated setting. Moreover, it contributes new insights into the tolerability of GDMT and the occurrence of AEs across different risk groups, especially AHF and other HF subpopulations. Our analysis identified two key factors as particularly relevant for GDMT up-titration, hyperkalemia and impaired kidney function, both of which were more common in high-risk patients. Notably, higher baseline eGFR and lower potassium levels emerged as strong predictors of successful up-titration. Importantly, modern evidence suggests that neither impaired kidney function nor HK should be considered insurmountable barriers to GDMT optimization, as both conditions can often be effectively managed.

HK is a common issue in heart failure, linked to the use of RASi and MRA, as well as severe HF, renal impairment, diabetes, and older age [27]. However, HK is now manageable with potassium-binding agents, enabling higher doses of RASi and MRAs [28,29]. Poor kidney function should not preclude GDMT, as studies like STOP-ACEi and the Swedish HF Registry show that continuing RASi and MRAs is safe, even in severe renal dysfunction [30,31]. Moreover, therapies like SGLT2i and ARNI stabilize renal function long-term [32,33]. Additionally, a recent study demonstrated benefits of ARNI treatment even in patients with end stage renal disease requiring dialysis [34]. These findings highlight the importance of maintaining GDMT despite HK or renal concerns to optimize outcomes in high-risk HF patients.

### 4.5. Limitations

This study has several limitations. The specialized HF outpatient setting may not reflect non-specialized care, limiting applicability to primary care or community hospitals. Potential confounders, such as patient adherence, patient requests, and multidisciplinary management, could influence treatment consistency and up-titration decisions. While the study emphasized safety and opportunity of GDMT up-titration and AEs, efficacy parameters like quality of life, functional improvement, and risk reduction are only assumptions from the respective outcome trials and metanalysis [24]. Additionally, evolving therapies, such as SGLT2i, Vericiguat, and potassium binders, were gradually implemented in our patient cohort, which may have impacted GDMT implementation and related factors during the study period (2015–2023).

## 5. Conclusions

This study demonstrates that GDMT can be successfully implemented at much higher rates than reported in previous registries, especially among AHF. Importantly, AEs are generally infrequent and mainly not be directly attributable to AHF. These findings emphasize that AEs, though more common in high-risk patients, should not deter GDMT up-titration. Further research is needed to establish stronger evidence on the safety, tolerability, and especially the benefits of GDMT up-titration in AHF.

## Figures and Tables

**Table 1:** Baseline characteristics according to NT-proBNP levels.

Baseline Characteristics	Total Cohort	NT-proBNP < 1000 pg/mL	NT-proBNP 1000–2000 pg/mL	NT-proBNP > 2000 pg/mL	*p*-Value
Basic demographics					
Age, years (IQR)	62 (50–72)	57 (48–64)	62 (49–70)	66 (54–73)	<0.001
Male sex, n (%)	286 (76.7%)	70 (76.1%)	63 (80.8%)	153 (75.4%)	0.637
BMI, kg/m^2^ (IQR)	26.7 (23.4–30.8)	28.4 (24.9–31.1)	26.6 (23.2–30.4)	26.2 (23.2–30.8)	0.103
Systolic BP, mmHg (IQR)	124 (110–140)	125 (113–135)	120 (114–141)	124 (108–140)	0.531
Diastolic BP, mmHg (IQR)	80 (70–89)	79 (70–85)	80 (72–90)	78 (70–90)	0.502
Heart rate, bpm (IQR)	74 (65–85)	68 (60–77)	74 (64–85)	76 (67–88)	<0.001
NYHA class, n (%) †					
I	29 (7.8%), n = 370	16 (17.4%)	5 (6.4%)	8 (4.0%)	<0.001
II	191 (51.6%), n = 370	58 (63.0%)	43 (55.1%)	90 (45.0%)	
III	145 (39.2%), n = 370	18 (19.6%)	30 (38.5%)	97 (48.5%)	
IV	5 (1.4%), n = 370	-	-	5 (2.5%)	
Comorbidities					
Arterial hypertension, n (%)	176 (47.2%)	38 (41.3%)	37 (47.4%)	101 (49.8%)	0.397
CAD, n (%)	175 (46.9%)	35 (38%)	41 (52.6%)	99 (48.8%)	0.120
Atrial fibrillation, n (%)	168 (45.0%)	30 (32.6%)	36 (46.2%)	102 (50.2%)	0.018
Diabetes mellitus type II, n (%)	146 (39.1%)	26 (28.3%)	30 (38.5%)	90 (44.3%)	0.031
Chronic kidney disease, n (%)	186 (49.9%)	20 (21.7%)	35 (44.9%)	131 (64.5%)	<0.001
COPD, n (%)	44 (11.8%)	7 (7.6%)	9 (11.5%)	28 (13.8%)	0.319
PAD, n (%)	48 (12.9%)	4 (4.3%)	11 (14.1%)	33 (16.3%)	0.010
Carotid artery disease, n (%)	24 (6.4%)	3 (3.3%)	5 (6.4%)	16 (7.9%)	0.366
Stroke, n (%)	24 (6.4%)	6 (6.5%)	3 (3.8%)	15 (7.4%)	0.636
Iron deficiency, n (%) †	147 (50.2%), n = 293	26 (32.1%)	32 (54.2%)	89 (58.2%)	0.001
Any malignant disease, n (%)	57 (15.3%)	10 (10.9%)	7 (9.0%)	40 (19.7%)	0.035
Medication					
Beta-blocker, n (%)	347 (93%)	85 (92.4%)	72 (92.3%)	190 (93.6%)	0.890
Target dose ≥ 50%, n (%)	243 (65.1%)	65 (70.7%)	45 (57.7%)	113 (65.5%)	0.214
RASi, n (%)	331 (88.7%)	86 (93.5%)	73 (93.6%)	172 (84.7%)	0.031
Target dose ≥ 50%, n (%)	202 (54.2%)	63 (68.5%)	51 (65.4%)	88 (43.3%)	<0.001
MRA, n (%)	271 (72.7%)	72 (78.3%)	63 (80.8%)	136 (67.0%)	0.028
Target dose ≥ 50%, n (%)	264 (70.8%)	71 (77.2%)	61 (78.2%)	132 (65.0%)	0.030
SGLT2i, n (%) *	85 (52.8%)	22/37 (59.5%)	20/42 (47.6%)	43/82 (52.4%)	0.558
Diuretics, n (%)	212 (56.8%)	49 (53.3%)	51 (65.4%)	112 (55.2%)	0.221
Devices					
ICD, n (%)	144 (38.6%)	29 (31.5%)	35 (44.9%)	80 (39.4%)	0.203
CRT, n (%)	89 (23.9%)	19 (20.7%)	21 (26.9%)	49 (24.1%)	0.622
Laboratory parameters					
NT-proBNP pg/mL (IQR)	2363 (1014–5009)	508 (378–707)	1430 (1216–1719)	4730 (3199–8307)	<0.001
Creatinine, mg/dL (IQR)	1.17 (0.96–1.57)	0.98 (0.81–1.12)	1.16 (0.99–1.35)	1.39 (1.06–2.04)	<0.001
eGFR, ml/min/1.73 m^2^ (IQR)	60.23 (42.55–77.26)	74.99 (63.29–89.29)	62.74 (48.75–77.06)	47.57 (31.47–68.01)	<0.001
Potassium, mmol/L (IQR)	4.73 (4.40–5.03)	4.69 (4.36–5.03)	4.77 (4.44–5.11)	4.71 (4.39–4.99)	0.257
BUN, mg/dL (IQR) †	22.1 (16.3–33.1), n = 339	17.4 (13.9–23.7)	19.2 (15.8–28.1)	27.1 (18.9–38.7)	<0.001

BMI = Body Mass Index; BP = Blood Pressure; CRT = Cardiac Resynchronization Therapy; eGFR = estimated Glomerular Filtration Rate; ICD = Implantable Cardioverter Defibrillator; MRA = Mineralocorticoid Receptor Antagonist; NT-proBNP = N-terminal-pro-Brain Natriuretic Peptide; NYHA = New York Heart Association; RASi = Renin–Angiotensin System Inhibitor; SGLT2i = Sodium–Glucose-Transporter2-Inhibitor; * SGLT2i data after 2021, in alignment with the ESC recommendations for SGLT2i use. † indicates variables with missing data, and the number of complete cases is specified.

**Table 2:** Logistic regression model for up-titrational success at 12 months.

Variable	ß-Coefficient	Standard Error	T-Value	*p*-Value	Odds Ratio	95% CI	Cox and Snell Pseudo R^2^
Intercept	−0.298						0.150
eGFR (baseline) *	0.679	0.143	4.733	<0.001	1.972	1.488–2.612	
Triple-therapy TD (baseline) *	0.523	0.118	4.432	<0.001	1.686	1.338–2.125	
Potassium (baseline) *	−0.240	0.121	1.975	0.048	0.787	0.620–0.998	

* Variables are z-transformed, increase as per standard unit.

**Table 3:** Reasons for suboptimal GDMT (TD ≤ 50%) at 12 months.

Reasons	BB TD ≤ 50% (n = 86/373)	RASi TD ≤ 50% (n = 84/373)	MRA TD ≤ 50% (n = 141/373)
Asymptomatic hypotension	16/86 (18.6%)	17/84 (20.2%)	-
Bradycardia	32/86 (37.2%)	-	-
Hyperkalemia	-	26/84 (30.9%)	61/141 (43.2%)
Impaired renal function	-	8/84 (9.5%)	12/141 (8.5%)
Hyperkalemia and impaired renal function	-	6/84 (7.1%)	21/141 (14.9%)
Dizziness	3/86 (3.5%)	1/84 (11.9%)	-
Symptomatic hypotension	9/86 (10.5%)	8/84 (9.5%)	-
Symptomatic bradycardia	3/86 (3.5%)	-	-
Syncope/presyncope	2/86 (2.3%)	-	-
Intolerance/patient request/allergy	6/86 (6.9%)	3/84 (3.5%)	6/141 (4.3%)
Medication discontinued elsewhere	8/86 (9.3%)	7/84 (8.3%)	5/141 (3.5%)
Total number of reasons	79/86 (91.9%)	76/84 (90.5%)	105/141 (74.5%)
No reason	7/86 (8.1%)	8 (9.5%)	36 (25.5%)

## Data Availability

The data underlying this article cannot be shared publicly due to ethical and privacy concerns regarding the individuals who participated in the study. The data will be shared on reasonable request to the corresponding author.

## Abbreviations

The following abbreviations are used in this manuscript:

ACEi	Angiotensin-Converting Enzyme Inhibitor
AHF	Advanced Heart Failure
ARB	Angiotensin Receptor Blocker
ARNI	Angiotensin Receptor-Neprilysin Inhibitor
BB	Beta-blocker
eGFR	Estimated Glomerular Filtration Rate
GDMT	Guideline Directed Medical Therapy
HF	Heart Failure
HFA	Heart Failure Association
HFrEF	Heart Failure with reduced Ejection Fraction
HK	Hyperkalemia
IQR	Interquartile Range
LVEF	Left Ventricular Ejection Fraction
MRA	Mineralocorticoid Receptor Antagonist
NYHA	New York Heart Association
RASi	Renin Angiotensin System Inhibitor
RHR	Resting Heart Rate
SGLT2i	Sodium–Glucose Transporter 2 Inhibitor
